# Supplementary material for: Learning curve and surgical outcome of robotic assisted colorectal surgery with ERAS program
Source: Sci Rep. 2022 Nov 29;12:20566. doi: 10.1038/s41598-022-24665-w (PMC9709162; doi:10.1038/s41598-022-24665-w)
Supplement: Supplementary file 2 — Supplementary Information 2. [file 41598_2022_24665_MOESM2_ESM.zip › RAL Raw data-Ñ[▒K/113/OP N_ñw╝╨░O▒Kñσ.pdf]

W52 / W52

臺中榮民總醫院
手術室護理記錄

[redacted]

基本資料

- 室別： 開刀房-17室，手術科別： CRS，手術類別： 常規，日期： 2021/09/23
- 進入時間： 0725 ； 麻醉開始時間： 0745 ； Time Out時間： 0930 ； 劃刀時間： 0931 ； 手術結束時間： 1500 送出時間： 1530 ；
- 麻醉方式： GE
  診斷：S COLON CA
  手術名稱：Robot Assisted Colectomy(L)
  手術局部用藥：NIL
  術前預防性抗生素：
  - 抗生素名稱一： Veterin 1GM IVD/由麻醉護理師給予
  - 時間： 0905
  - 上次簽章: 陳盈妤
  ，追加預防性抗生素：
  - 抗生素名稱一： Veterin 1GM IVD/由麻醉護理師給予
  - 時間： 1335
  - 上次簽章: 歐真勤
- 手術醫師： 蔣鋒帆，陳映菁，林志安
- 麻醉醫師： VS 陳永銘
-
  - 刷手護士A ： 黃彥翔/劉雅汶，時間： 0725，刷手護士B ： 歐真勤，時間： 0725-1100，接替刷手一： 黃彥翔/劉雅汶，時間： 1200，接替刷手二： 陳盈妤/V，時間： 1500
  - 流動護士A ： 陳盈妤，時間： 0725，接替流動一： 歐真勤，時間： 1200，接替流動二： 許芷瑄，時間： 1500

皮膚狀況

A-V Shunt： 無
術前： 完整
記錄者： 上次簽章: 陳盈妤
術後： 完整
記錄者： 許芷瑄

手術準備

手術臥式 單一手術臥式

-
  - 手術臥式： 截石臥式，NIL
  - 使用附件： 溫毯 40 ℃，其它： 海綿墊
  - 皮膚準備： Hibiscrub，2% Chlorhexidine，其它： NIL
  - 電燒： 使用電燒: 電極片位置： 其它： 導電軟墊，雙極電燒，記錄者： 上次簽章: 陳盈妤

止血帶T： 無

植入物

無

標本送檢

- 細菌培養 無
- 細胞學 無
- 冰凍切片 無
- 病理標本
  組織名稱一：S colon
  ,
  組織名稱二：distal cut end
  ,

組織名稱三： IMA root

- 截肢 無

標本交付 標本： 3 袋 ，標本： 0 盒 ，標本： 0 瓶 ，細胞學： 0 管 ，Culture Tube： 0 管 ，被交付醫師： R 林志安，

- 記錄者： 上次簽章: 歐真勤
- 時間： 1430

傷口術後敷料

傷口敷料： 傷口敷料： 無

- 請於圖上標示部位：

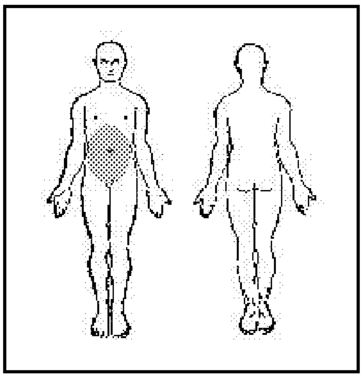

- 傷口敷料： 紗布

導管

導管	天數	導管資訊	插入日期	插入時間	更換日期	拔除日期	拔除時間	拔除原因	預計拔除/更換	預計拔除/更換日期
Foley(1)	1	號碼:16 Fr.; 部位:膀胱;	2021/09/23	0810						
A-line (1)	1	號碼:20G ; 部位:左手;	2021/09/23	0812						
Penrose Drain(1)	1	號碼:12 mm.; 部位:骨盆腔;	2021/09/23	1330						
Sump NG Tube(1)	1	號碼:16 Fr.; 部位:胃; 註記:fix 55cm;	2021/09/23	0815						
Epidural PCA(1)	1	部位:硬脊膜外;	2021/09/23	0840						
Double Lumen CVC(1)	1	號碼:7Fr. ; 部位:; 象限:RU; 註記:fix 15cm;	2021/09/23	0805						

用物計數

- 手術中出血量： 950 c.c.
- 手術中尿量： 800 c.c.
-
    - 其它： 無
    - NIL

用物計數： 多切口同時縫合

- 1.器械： 正確，2.縫針： 正確，3.刀片： 正確，4.紗布： 正確，5.腹部墊： 未使用，6.紗球： 未使用，7.PEANUTS ： 未使用，8.紗條： 未使用，9.絲帶： 未使用，10.針頭： 未使用，11.Vessel loop： 未使用，12.Boots： 未使用，13.Jelco： 未使用，14.棉條： 未使用，
    - 第一次：
        - 刷手護士 ： 黃彥翔/劉雅汶
        - 流動護士： 上次簽章: 歐真勤
        - 時間： 1330

,

    - 第二次：
        - 刷手護士 ： 黃彥翔/劉雅汶
        - 流動護士： 上次簽章: 歐真勤
        - 時間： 1350

,

- 第三次：
  - 刷手護士 ： 黃彥翔/劉雅汶
  - 流動護士： 上次簽章: 歐真勤
  - 時間： 1400

備註

備註： davinci 30度 XI scopy:No.1

- 記錄者： 上次簽章: 陳盈妤
- 時間： 0805

填寫護理人員 許芷瑄

正式摘要

W52 / W52

doctorRank:B
